# RNAi-Mediated PD-L1 Inhibition for Pancreatic Cancer Immunotherapy

**DOI:** 10.1038/s41598-019-41251-9

**Published:** 2019-03-18

**Authors:** Byunghee Yoo, Veronica Clavijo Jordan, Patrick Sheedy, Ann-Marie Billig, Alana Ross, Pamela Pantazopoulos, Zdravka Medarova

**Affiliations:** 1 MGH/MIT/HMS Athinoula A. Martinos Center for Biomedical Imaging, Massachusetts General Hospital and Harvard Medical School, Boston, MA 02129 USA; 2 Department of Health Sciences, CaNCURE Program, Northeastern University, Boston, MA 02115 USA

## Abstract

The recent past has seen impressive progress in the treatment of various malignancies using immunotherapy. One of the most promising approaches involves immune checkpoint inhibitors. However, the clinical results with these agents have demonstrated variability in the response. Pancreatic cancer, in particular, has proven resistant to initial immunotherapy approaches. Here, we describe an alternative strategy that relies on combining gemcitabine and a novel programmed death-ligand 1 (PD-L1) inhibitor, termed MN-siPDL1. MN-siPDL1 incorporates small interfering RNA against PD-L1 (siPDL1) conjugated to a magnetic nanocarrier (MN). We show that noninvasive magnetic resonance imaging (MRI) could be used to monitor therapeutic response. Combination therapy consisting of gemcitabine and MN-siPDL1 in a syngeneic murine pancreatic cancer model resulted in a significant reduction in tumor growth and an increase in survival. Following optimization, a 90% reduction in tumor volume was achieved 2 weeks after the beginning of treatment. Whereas 100% of the control animals had succumbed to their tumors by week 6 after the beginning of treatment, there was no mortality in the experimental group by week 5, and 67% of the experimental animals survived for 12 weeks. This method could provide therapeutic benefit against an intractable disease for which there are no effective treatments and which is characterized by a mere 1% 5-year survival.

## Introduction

Pancreatic cancer is the fourth-leading cause of cancer-related death in the United States with an overall 5-year survival rate of only 8%^1^. Surgical resection remains the treatment of choice for patients with resectable disease. However, less than 20% of the diagnosed patients qualify for curative resections^2^, 30% of patients present with regional disease, and 50% present with distal metastases^3^ with survival rates of 11% and 2%, respectively^1^. The reasons behind such poor prognosis have been postulated to involve the advanced stage at the time of diagnosis^2^, and resistance to standard chemotherapies^4^. There are multiple factors that are conceived to confer chemo-resistance: the formation of desmoplastic stroma limiting drug delivery, the activation of pancreatic stellate cells by reactive oxygen species, cytokines, and/or growth factors, and activated stellate cell secretion of immunosuppressive signaling molecules^4,5^. Due to the complex tumor biology of pancreatic cancer, multiple combination chemotherapies have emerged. As such, FOLFIRINOX (a combination consisting of 5-fluorouracil, leucovorin, irinotecan, and oxaliplatin), and gemcitabine/nab-paclitaxel have shown improvements in overall survival compared to standard gemcitabine monotherapy treatment^6,7^. However, these combination therapies are heavily dependent on the patient’s overall health, and the overall survival benefit for the latest cytotoxic combination therapies is only ~ 2–5 months.

In light of the tremendous suffering caused by this disease and the modest progress achieved thus far with cytotoxic treatments, it is clear that we need to explore radical, transformative approaches for therapy that attack the disease from multiple angles.

The last decade has seen tremendous progress in the field of cancer immunotherapy. In fact, immunotherapy represents the most promising new cancer treatment approach since the development of the first chemotherapies in the 1940s. Checkpoint inhibitors have worked against lethal cancers such as melanoma and some lung cancers – sometimes with dramatic success – and are being tested in dozens of other cancer types^8,9^. But pancreatic cancer has proven difficult to treat with conventional drugs and has been resistant to initial immunotherapy approaches. Partly, the reason for this is the complex tumor microenvironment that characterizes pancreatic adenocarcinoma. Chiefly, the presence of desmoplastic tumor stroma that is both immunosuppressive in nature and a physical barrier for antibody and T lymphocytes infiltration^10^. Consequently, it is important to design alternative approaches that combine: innovative checkpoint inhibitors that can be delivered efficiently to tumor cells and tumor resident macrophages, and strategies that enhance the permeation of the tumor by T lymphocytes.

Here, we explore an alternative strategy that relies on combining gemcitabine (Gem) and a novel PD-L1 inhibitor (termed MN-siPDL1). MN-siPDL1 incorporates a nanoparticle carrier that is delivered with high efficiency to tumor cells *in vivo*^11–19^, where it post-transcriptionally inhibits PD-L1 expression on tumor cells via the RNA interference mechanism. The approach is advantageous over small molecules or antibodies because the siRNA component inhibits the target antigen at the post-transcriptional level and not at the protein level. Also, the RNAi mechanism is catalytic and necessitates the delivery of only picomolar amounts of siRNA to the tumor cell for the abolition of the target antigen. By contrast, small molecules or antibodies require the achievement of at least a 1:1 molar ratio of antigen to therapeutic molecule and could be ineffective in the presence of a compensatory increase in the expression of the target antigen by the tumor cell.

A key advantage of our therapeutic approach also derives from the fact that MN-siPDL1 incorporates a superparamagnetic nanoparticle core whose accumulation in tissues over time could be monitored by noninvasive MRI. This capability is highly significant when designing and optimizing therapeutic protocols in the process of drug development.

In the current study, we administered 7 weeks of combination therapy consisting of gemcitabine and MN-siPDL1 in a syngeneic murine pancreatic cancer model. This approach resulted in significantly lower morbidity and toxicity, leading to tumor regression and a dramatic improvement in survival. In particular, following dose optimization, a 90% reduction in tumor volume was achieved 2 weeks after the beginning of treatment. Whereas 100% of the control animals had succumbed to their tumors by week 6 after the beginning of treatment, there was no mortality in the experimental group by week 5, and 67% of the experimental animals survived for 12 weeks.

## Methods

### Small interfering RNA (siRNA) oligos

The sequence of the siRNA oligo against *pd-l1* (siPDL1; MW = 13,788.9 g/mol), consisted of 5′-ThioMC6-D/GGUCAACGCCACAGCGAAUUU-3′ (sense sequence) and 5′-PAUUCGCUGUGGCGUUGACCUU-3′ (anti-sense sequence). The sequence of the scrambled siRNA oligo (siSCR; MW = 13,728.8 g/mol) was 5′-ThioMC6-D/UGGUUUACAUGUCGACUAAUU-3′ (sense sequence) and 5′-PUUAGUCGACAUGUAAACCAUU-3′ (anti-sense sequence). Both siRNAs were designed and synthesized by Dharmacon (Lafayette, CO). The 5′-Thiol-Modifier C6 disulfide (5′-ThioMC6) was introduced into the sense sequences in order to permit conjugation to the magnetic nanoparticles.

### Synthesis of dextran coated magnetic nanoparticles (MN)

MN was synthesized following a protocol published previously^20^. Briefly, 30 ml of Dextan-T10 (0.3 g ml^−1^, Pharmacosmos A/S, Holbaek, Denmark) was mixed with 1 ml of FeCl_3_.6H_2_O (0.65 g ml^−1^, Sigma, Saint Louis, MO) while flushing Argon gas for an hour. 1 ml of FeCl_2_.4H_2_O (0.4 g ml^−1^, Sigma) was added into the mixture. Following, 15 ml of cold NH_4_OH (28%, Sigma) was added dropwise to the stirred mixture. The temperature was increased to 85 °C for 1 h to start the formation of a nanoparticulate dispersion and then cooled to room temperature. The magnetic nanoparticles were concentrated to 20 ml using Amicon ultra centrifugal units (MWCO 30 kDa; Millipore, Darmstadt, Germany). The resulting dextran-coated magnetic nanoparticles were cross-linked by epichlorohydrin (14 ml, 8 h, Sigma) and aminated by the addition of NH_4_OH (28%, 60 ml). Aminated magnetic nanoparticles (MN) were purified by dialysis and concentrated using Amicon ultra centrifugal units. The properties of MN were as follows: concentration, 10.94 mg ml^−1^ as Fe; the number of amine groups per MN, 64; relaxivity (R2), 82.5 ± 1.16 mM^−1^sec^−1^; size of MN, 20.3 ± 0.6 nm (NanoSight LM-10 system and Nanoparticles Tracking Analysis software (Ver. 3.2), Malvern, UK).

### Nanodrug Synthesis and Characterization

Nanodrug synthesis was carried out according to a previously published protocol^20^. Briefly, MN was conjugated to the heterobifunctional linker N-Succinimidyl 3-[2-pyridyldithio]-propionate (SPDP, Thermoscientific Co., Rockford, IL), which was utilized for the linkage of activated siRNA oligos. SPDP was dissolved in anhydrous DMSO and incubated with MN. The 5′-ThioMC6 of the siRNA oligo was activated to release the thiol via 3% TCEP (Thermoscientific Co., Rockford, IL) treatment in nuclease free PBS. The siRNA oligos were purified using an ammonium acetate/ethanol precipitation method. After TCEP-activation and purification, each oligo (siPDL1 and siSCR) was dissolved in water and incubated with the SPDP modified MN overnight to prepare the final product (MN-siPDL1 and MN-siSCR). Oligos were added to MN at two different ratios to obtain nanodrugs incorporating 2.1 ± 0.4 (low-dose) or 4.8 ± 0.7 (high-dose) siRNA oligos per MN, as quantified by electrophoresis^20^. The size of the final MN-siPDL1/SCR was 23.2 ± 0.9 nm.

### Cell lines

The murine pancreatic ductal adenocarcinoma, PAN 02 cell line (NCI, Frederick, MD) was cultured in RPMI 1640 culture medium (Sigma), supplemented with 10% FBS (Thermoscientific, Waltham, MA), 1% antibiotics (Invitrogen, Carlsbad, CA), and 2 mM L-glutamine, per the supplier’s instructions. The medium was changed 3 times per week and trypsinized for sub-culturing once per week.

### Immunohistological Tissue staining and Fluorescence Microscopy

Primary and secondary antibodies were purchased from Abcam (Cambridge, MA) and included: anti-CD3 (Cat. #: AB16669), anti-CD8 (Cat. #: AB25478), anti-FoxP3 (Cat. #: AB75763), Granzyme B (Cat. #: AB4069), anti-Ki67 (Cat. #: AB16667), and anti-PDL1 (Cat. #: AB80276) as primary antibodies, Goat Anti-Rat IgG H&L (DyLight 488 pre-adsorbed, AB98420) and Goat Anti-Rabbit IgG H&L (DyLight 488 pre-adsorbed, AB96899) as secondary antibodies.

The immunohistological tissue staining was performed following the protocol for each biomarker. Briefly, excised tumor tissues were embedded in Tissue-Tek OCT compound (Sakura Finetek, Torrance, CA) and snap frozen in liquid nitrogen. The tissues were cut into 7 µm sections and fixed in 4% formaldehyde for 10 min. Detergent permeabilization was performed using 0.1% Triton X-100 in PBS, when needed. After blocking with 5% goat serum in 0.5% bovine serum albumin in PBS, each slide was incubated with corresponding primary antibody (dilution 1/200) at 5 °C overnight. Each slide was incubated with secondary antibody (dilution 1/200) for 60 min and mounted with Vectashield mounting medium with DAPI (Vector Laboratories, Inc. Burlingame, CA). The slides were analyzed using a Nikon E400 fluorescence microscope (Nikon, Tokyo, Japan), equipped with the necessary filter sets (MVI Inc., Avon, MA). Images were acquired using a charge coupled device camera with near-IR sensitivity (SPOT 7.4 Slider RTKE; Diagnostic Instruments, Sterling Heights, MI). The images were analyzed using SPOT 4.0 Advance version software (Diagnostic Instruments) and ImageJ (Ver. 1.51c, NIH).

### *In vivo* MR Imaging

MR imaging was performed before and after each weekly treatment with MN-siPDL1/SCR using a Bruker 9.4 T horizontal bore magnet (Magnex Scientific) with gradient insert and operated using ParaVision 5.1 software. Mice formed tumors 2 weeks after inoculation and were monitored by MR imaging for the quantitative measurement of tumor volumes, utilizing a rapid acquisition with relaxation enhancement (RARE) T_1_ weighted protocol (TE = 8.5 msec, TR = 2500 msec, number of average = 4, RARE factor = 4, FOV = 4 × 4 cm^2^, matrix size = 128 × 128 pixels, number of slices = 50, slice thickness = 0.5 mm, and interslice thickness = 0.5 mm) and T_2_ weighted protocol (TE = 8.5 msec, TR = 7500 msec, number of averages = 4, RARE factor = 16, FOV = 4 × 4 cm^2^, matrix size = 128 × 128 pixels, number of slices = 50, slice thickness = 0.5 mm, and interslice thickness = 0.5 mm, flip angle = 162 degrees). Multi slice multi-echo (MSME) T2-weighted maps were collected with the following parameters: TE = 8, 16, 24, 32, 40, 48, 56, 64, 72, and 80 msec, TR = 4500 msec, number of slices = 5, slice orient = axial, number of averages = 1, RARE factor = 1, field of view = 4 × 4 cm^2^, matrix size = 128 × 128 pixels, slice thickness = 0.5 mm, interslice thickness = 0.5 mm, flip angle = 128 degrees). The measurement of tumor volumes and the reconstructions of the T2 maps were performed by two independent investigators blinded to sample identity to account for variability in region of interest (ROI) selection. T2 maps and T2 relaxation times in the tumors were calculated using ImageJ software (Ver. 1.50c, NIH). The relaxation rate R2 was obtained as a reciprocal of relaxation time. Delta R2 was calculated using the following equations: S = S_0_ Exp(TE/T_2_), ΔR2 = 1/T_2,1_− 1/T_2,2_ = (1/TE)*ln(S_1_/S_2_), and correlated to the concentration of MN following the equation: ΔR2 = r2•Δ[C]. All delta R2 values were calculated relative to week 0.

### Animal model

Six-week-old female mice (C57Bl/6) were implanted into the right flank with the murine pancreatic cancer cell line, Pan02 (0.25 × 10^6^ cells). One week after cell injection, tumor size was monitored by caliper measurements. Tumor volume was calculated according to the equation: Volume = 0.5 x L x W^2^, where L is length, and W, width. Treatment was initiated once tumor volumes reached 50 mm^3^, as estimated using calipers. Thereafter, tumor volume was measured by MRI once mice were enrolled in the study and before and after each weekly treatment. All animal experiments were performed in compliance with institutional guidelines and approved by the Institutional Animal Care and Use Committee at Massachusetts General Hospital.

### Therapy

Mice were randomly assigned to two experimental groups treated with low-dose MN-siPDL1 (10 mg kg^−1^ as iron; 520 nmoles/kg siRNA) in solution with gemcitabine (333.3 mg/kg)(n = 6), or high-dose MN-siPDL1 (10 mg kg^−1^ as iron; 937 nmoles/kg siRNA) in solution with gemcitabine (333.3 mg/kg)(n = 6) and two control groups treated with MN-siSCR + gemcitabine at the same doses (n = 6). Experiments were performed in three independent trials. The drugs were administered weekly as a mixture of nanodrug and gemcitabine (i.v.). After week 7, co-administration of gemcitabine was discontinued to avoid exceeding the maximum tolerated dose, and only nanodrug was administered until the end of the study. All mice were monitored weekly by magnetic resonance imaging to keep track of changes in tumor volume for a maximum of 12 weeks after the first treatment or until animals became moribund.

### Statistical analysis

Data were expressed as mean ± s.d. or s.e.m., where indicated. Statistical comparisons were drawn using a two-tailed t-test (SigmaStat 3.0; Systat Software, Richmond, CA). A value of P < 0.05 was considered statistically significant.

## Results

### MN-siPDL1 can be delivered *in vivo* to primary tumors and the delivery can be monitored by noninvasive MRI

**Figure 1:**
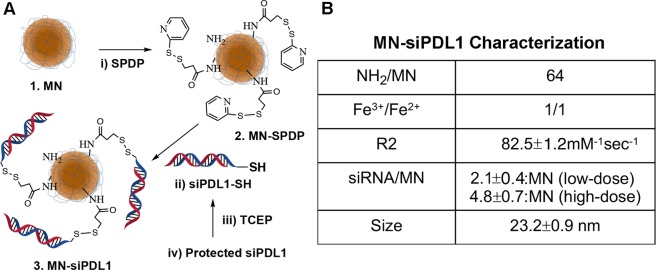
Structure, synthesis, and characterization of MN-siPDL1. (**A**) MN-siPDL1 was synthesized by sequential conjugation of 20-nm aminated dextran-coated superparamagnetic iron oxide nanoparticles to the heterobifunctional labile linker SPDP and siRNA against PD-L1. (**B**) MN-siPDL1 characterization.

In order to successfully deliver therapeutic amounts of siRNA to tumor cells following intravenous injection, we needed to optimize the design of MN-siPDL1 in terms of hydrodynamic size, conjugation method, and number of siRNA oligos per nanoparticle. The synthetic scheme of MN-siPDL1 is illustrated in Fig. 1A. To ensure long circulation times (>4 hrs.) and efficient diffusion across the vascular endothelium and throughout the tumor interstitium, we designed MN-siPDL1 so that its final size after sequential conjugation to the SPDP linker and the oligo was 23.2 ± 0.9 nm. The number of siRNA oligonucleotides per nanoparticle was adjusted to no more than 5.5 with the goal of minimizing steric interference with bioconjugation. This design is optimized to enhance the uptake of the nanodrug by tumor tissue through the Enhanced Permeation-Retention (EPR) effect. In addition, the SPDP linker was chosen due to its reducible nature, which ensures dissociation of the oligo from the nanoparticle in cancer cells and efficient entry into the RNA-induced silencing complex (RISC). Finally, to permit detection of MN-siPDL1 by magnetic resonance imaging, the relaxivity (R2) of the final preparation was adjusted by varying the ratios of [Fe^3+^]/[Fe^2 +^] to achieve an R2 of 82.5 ± 1.16 mM^−1^sec^−1^ (Fig. 1B).

For the purposes of establishing an effective therapeutic protocol, we needed to confirm delivery of MN-siPDL1 to pancreatic tumor tissue and to demonstrate the capability of MRI to semi-quantitatively measure MN-siPDL1 bioavailability in tumors. We tested our hypothesis using the PAN02 syngeneic pancreatic cancer model. These animals formed tumors 2 weeks after inoculation and were monitored by MR imaging, using single-echo and multi-echo T_2_ weighted protocols.

**Figure 2:**
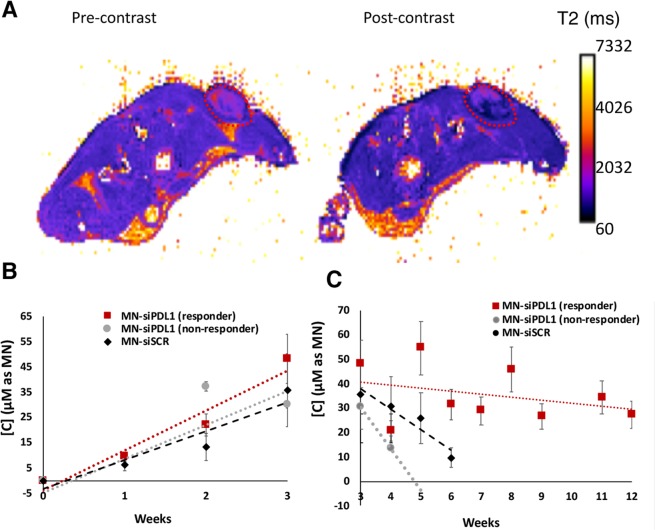
Image-guided delivery of MN-siRNA to tumors. (**A**) T2 maps of tumor bearing animals. The localization of MN-siPDL1 in tumor tissue caused shortening of the T2 relaxation time and resulted in negative contrast as compared to the pre-contrast image. (**B**) MN-siPDL1 concentration measurements over the tumor region-of-interest (ROI) in experimental and control animals during the first 3 weeks of treatment. The accumulation rate of MN-siPDL1 was faster than that of MN-siSCR during the first three weeks of treatment. The accumulation rate of MN-siPDL1 in non-responder animals was intermediate. (**C**). MN-siPDL1 concentration measurements over the tumor region-of-interest (ROI) in experimental and control animals during weeks 3–12 of treatment. The concentration of the agent in the control group treated with MN-siSCR or in non-responder animals treated with MN-siPDL1 decreased faster than in the responder animals treated with MN-siPDL1.

As shown in Fig. 2A, the localization of MN-siPDL1 in tumor tissue caused shortening of the T2 relaxation time and resulted in negative contrast as compared to the pre-contrast image. The delta R_2_- derived concentration of MN-siPDL1 showed a linear increase during the first three weeks. The accumulation rate of MN-siPDL1 was 1.5-fold faster than that of MN-siSCR during that time period (Fig. 2B). Since the concentration of the nanodrug in tumor cells reflects mostly dilution due to cell division, the faster growth rate of the control tumors treated with MN-siSCR likely led to the observed slower increase in concentration over time in this group. This difference was more pronounced at the later stages of tumor growth, further supporting this hypothesis. The rate of concentration decrease, reflective of rapid nanodrug dilution due to tumor cell division in the control group treated with MN-siSCR, was 5.1-fold greater than in the experimental group treated with MN-siPDL1, indicating a more rapid growth of the tumor in the control animals (Fig. 2C).

Interestingly, a subgroup of animals treated with the high-dose MN-siPDL1 failed to respond to treatment, as defined by rapid tumor progression and limited survival. In that group, the early increase in tissue concentration of the MN label as measured by delta-R_2_ was intermediate between the animals that responded to treatment and those treated with MN-siSCR (Fig. 2B). At the later time points, the dilution of the MN label in that group was also significantly more rapid than in the responder animals (Fig. 2C). This observation suggested that indeed, the described imaging approach could represent a useful biomarker for response stratification during treatment.

### Combination treatment with gemcitabine and MN-siPDL1 is effective in a model of syngeneic pancreatic cancer

Our therapeutic studies illustrated the potential of the combination treatment with gemcitabine and MN-siPDL1 in pancreatic cancer. To determine whether treatment with gemcitabine in combination with MN-siPDL1 could inhibit tumor growth, the mice were treated with gemcitabine in solution with a low dose of MN-siPDL1 or siSCR (10 mg/kg Fe; 520nmoles/kg siRNA in both groups) or a high dose of MN-siPDL1 or siSCR (10 mg/kg Fe, 937nmoles/kg siRNA in both groups). The combination treatment was initiated when the tumor size reached >50 mm^3^ as measured by anatomical MR imaging and continued for 12 weeks. In all of the therapeutic studies, the change in tumor volume was monitored by anatomical MR imaging before the administration of each weekly treatment.

**Figure 3:**
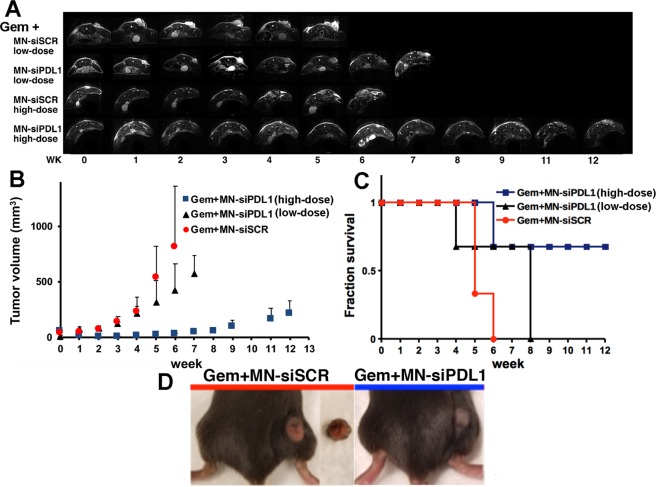
Combination treatment with gemcitabine and MN-siPDL1. (**A**) Representative T2-weighted MR images during the course of treatment. (**B**) Change in tumor volume during treatment. The response was significantly different between the high-dose active MN-siPDL1 and inactive MN-siSCR therapeutic in combination with gemcitabine beginning as early as week 2. In the low-dose MN-siPDL1 group, this difference was significant after week 6. (**C**) Kaplan-Meier survival analysis demonstrating improvement in survival in animals treated with MN-siPDL1 + gemcitabine vs. MN-siSCR + gemcitabine. (**D**) Photographs of tumor-bearing mice at week 6 demonstrating necrosis and ulceration in the control tumors.

The mice co-treated with MN-siPDL1 and gemcitabine demonstrated significant inhibition of tumor growth, relative to the inactive MN-siSCR controls (P < 0.05). This difference was evident at week 2 from the beginning of treatment, when tumor volume had decreased from 52.8 ± 6.7 mm^3^ in week 0 to 5.3 ± 0.8 mm^3^ in week 2 (p = 0.012). The difference persisted for the duration of the study (p < 0.05). Tumor volumes in the low-dose group were not different from the MN-siSCR control until week 6 (Fig. 3A,B).

The advantage of the combination treatment was clearly seen when assessing animal survival (Fig. 3C). 67% of the mice treated with gemcitabine and MN-siPDL1 (high dose) survived until week 12. 67% of the mice treated with gemcitabine and MN-siPDL1 (low dose) survived until week 8. All of the control mice treated with MN-siSCR and gemcitabine succumbed by week 6.

Interestingly, all of the mice in the group treated with gemcitabine and MN-siSCR developed large necrotic tumors, presumably due to the high rate of tumor growth. Tumor necrosis and ulceration was not seen in the experimental animals (Fig. 3D).

**Figure 4:**
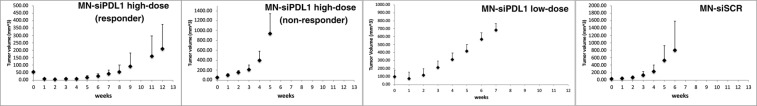
Combination treatment with gemcitabine and MN-siPDL1. Change in tumor volume during treatment for each of the treatment groups. The non-responders treated with a high-dose of MN-siPDL1 + gemcitabine had a tumor growth curve similar to the controls treated with MN-siSCR + gemcitabine.

Importantly, in the high-dose MN-siPDL1 cohort, a subgroup of the mice failed to respond and had tumor growth rate curves and survival that were analogous to the MN-siSCR group (Fig. 4). The time constants of tumor growth stratifying the experimental animals according to response are presented in Table 1. These results indicated variability of the response, warranting further investigation.

**Table 1:** Tumor Growth Rate Constants in Animals treated with MN-siPDL1 or MN-siSCR and Gemcitabine.

Treatment Group	Tumor growth rate constant (week^−1^)
High-dose MN-siPDL1 + gemcitabine (responder)	0.0813 ± 0.0502
High-dose MN-siPDL1 + gemcitabine (non-responder)	0.5427
Low-dose MN-siPDL1 + gemcitabine	0.3627 ± 0.0866
MN-siSCR + gemcitabine	0.5562 ± 0.24

### Combination treatment prevents the inactivation of cytotoxic T cells

**Figure 5:**
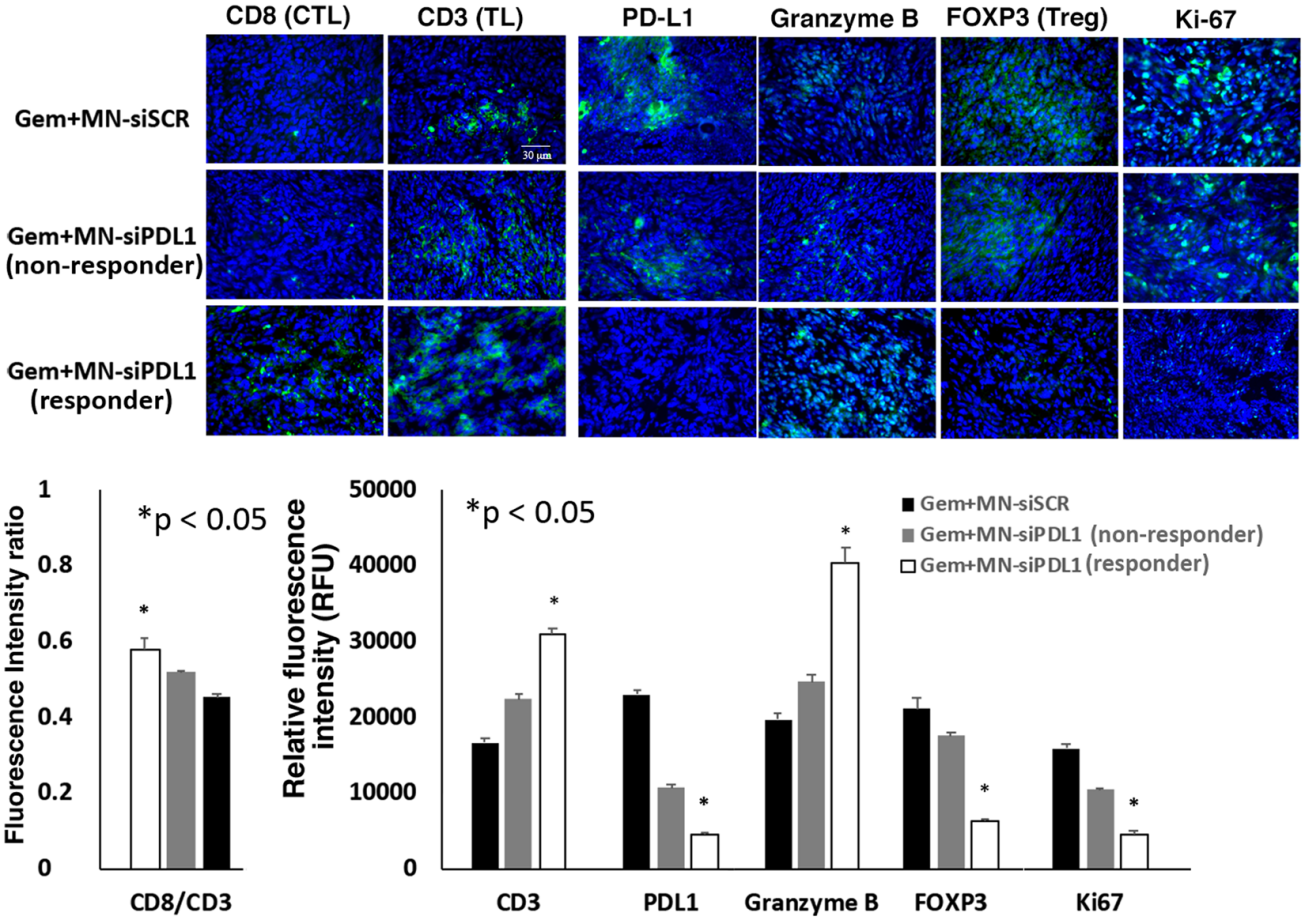
Immunofluorescence of tumors from mice treated with MN-siPDL1 and gemcitabine. (**A**) Representative micrographs and (**B**). Quantitative analysis of fluorescence signal intensity demonstrating efficient TIL recruitment, PD-L1 inhibition, cell-mediated cytotoxicity, Treg attenuation, and inhibition of tumor cell proliferation. TL: T lymphocytes; CTL: cytotoxic T lymphocytes; Treg: regulatory T cells; Ki-67: proliferation.

In order to assess the effect of treatment on the anti-tumor immune response, we analyzed tissue biomarkers of immune cell recruitment and activation in the tumors of treated mice. After combination treatment with MN-siPDL1 and gemcitabine, there was an increase in the recruitment of CD8+ tumor infiltrating lymphocytes (TILs). PD-L1 expression was significantly reduced. There was evidence of an increase in cell-mediated cytotoxicity, as evidenced by higher levels of Granzyme B and a decrease in the infiltration by immunosuppressive Foxp3 + regulatory T (Treg) cells. Finally, tumor cell proliferation was inhibited (Fig. 5). Interestingly, the expression of these biomarkers in non-responsive animals treated with high-dose MN-siPDL1 and gemcitabine, was intermediate between the control animals and the regressing experimental animals, suggesting that there is a critical level of PD-L1 inhibition needed in order to observe macroscopic response (Fig. 5). These results suggested that the observed therapeutic effect was the result of successful induction of an anti-tumor immune response.

## Discussion

Despite the promise of checkpoint inhibition for cancer immunotherapy, the response is generally variable, with a large number of patients failing to respond. Notable examples of FDA approved PD-L1 inhibitors include atezolizumab for metastatic non-small cell lung cancer (NSCLC)^21^ and durvalumab for locally advanced or metastatic urothelial carcinoma^22^. However, despite initial encouraging results and fast-track approval of atezolizumab for bladder cancer^23,24^, the confirmatory trial failed to achieve its primary endpoint of overall survival^25^. Similarly, a phase III trial of durvalumab with tremelimumab as a first-line treatment of non-small cell lung cancer failed to meet its primary endpoint of progression-free survival^26^. In pancreatic cancer, advances in checkpoint inhibitor-based therapies have shown disappointing clinical results. In a Phase II trial of the CTLA-4 inhibitor, Ipilimumab, monotherapy was ineffective with no responders resulting from the trial^27^. Similarly, in a multicenter Phase I trial an anti-PDL-1 antibody was administered intravenously in a variety of advanced cancer patients. Out of the 14 pancreatic cancer patients recruited, there were no objective responses reported^28^.

However, recent preliminary results from a randomized Phase II study in patients with metastatic pancreatic adenocarcinoma showed that combination therapy (Gemcitabine, Nab-Paclitaxel, Durvalumab, and Tremelimumab) was well tolerated with 73% of patients reporting partial response. Disease control rate was 100%, median progression free survival was 7.9 months, and 6-month survival was 80%^29^. These studies highlight the potential treatment efficacy of combination therapies with chemotherapy and checkpoint inhibitors.

Here, we describe an alternative design of a PD-L1 antagonist that post-transcriptionally inhibits PD-L1 expression on tumor cells via the RNA interference mechanism. The approach is advantageous over small molecules or antibodies because the siRNA component inhibits the target antigen at the post-transcriptional level and not at the protein level. Also, the RNAi mechanism is catalytic and necessitates the delivery of only picomolar amounts of siRNA to the tumor cell for the abolition of the target antigen. By contrast, small molecules or antibodies require the achievement of at least a 1:1 molar ratio of antigen to therapeutic molecule and could be ineffective in the presence of a compensatory increase in the expression of the target antigen by the tumor cell.

An additional key advantage of our therapeutic approach derives from the fact that it presents the unique opportunity to develop a clinically-relevant, image-guided treatment protocol that provides knowledge about therapeutic outcome, expressed both as change in tumor volume and tumor growth rate. The latter capability is made possible by the fact that MN-siPDL1 incorporates a 20-nm superparamagnetic nanoparticle carrier, which ensures highly efficient delivery to tumor cells and whose disposition in tissue over time can be monitored by quantitative noninvasive MRI. As suggested by our results, tumor delta-R2 would reflect tumor growth rate, which is expected based on the fact that the loss of these nanoparticles from tumor cells is governed by cell division. In addition to the assessment of tumor growth, anatomical MRI allowed the objective measurement of tumor volume as a morphologic biomarker of response. However, the application of dynamic MR imaging protocols could readily be used to also measure physiologic variables related to tumor blood flow and microvessel permeability.

In this sense, the described methodology represents an integrated tool for drug delivery and a synchronous biomarker of therapeutic response. Such tools, if introduced into the clinic, would genuinely exemplify the essence of rational precision medicine. Given that this approach will lay the foundation for future rational designs of novel therapies, we anticipate that further combinations of targets that may work synergistically by complementary mechanisms could be interesting. For example, one could envision combination therapies that physically alter the tumor microenviroment by enzymatic degradation via recombinant human hyaluronidase (PEGPH20)^30,31^, or other alternative chemotherapy agents, and/or alternative checkpoint inhibitors that may promote a synergistic effect in activating T-cells (PD-1 and CTLA-4). However, prior to expanding to alternative targets and combinations, the safety of the proposed approach will be tested in large animals as we prepare for Phase I testing.

On a more concrete level, while broadly applicable to solid malignancies, the present study focuses on pancreatic cancer because of its dismal prognosis and the lack of progress against its metastatic form. Approaches, such as the one described here could advance the treatment of pancreatic cancer and potentially vastly improve treatment outcomes in patients for whom no other therapeutic options are available.
